# Using event-related fMRI to examine sustained attention processes and effects of *APOE* ε4 in young adults

**DOI:** 10.1371/journal.pone.0198312

**Published:** 2018-06-01

**Authors:** Simon Evans, Devin Clarke, Nicholas G. Dowell, Naji Tabet, Sarah L. King, Samuel B. Hutton, Jennifer M. Rusted

**Affiliations:** 1 School of Psychology, University of Sussex, Brighton, East Sussex, United Kingdom; 2 School of Psychology, University of Surrey, Guildford, Surrey, United Kingdom; 3 Brighton and Sussex Medical School (BSMS), Brighton, East Sussex, United Kingdom; University of California, San Francisco, UNITED STATES

## Abstract

In this study we investigated effects of the *APOE* ε4 allele (which confers an enhanced risk of poorer cognitive ageing, and Alzheimer’s Disease) on sustained attention (vigilance) performance in young adults using the Rapid Visual Information Processing (RVIP) task and event-related fMRI. Previous fMRI work with this task has used block designs: this study is the first to image an extended (6-minute) RVIP task. Participants were 26 carriers of the *APOE* ε4 allele, and 26 non carriers (aged 18–28). Pupil diameter was measured throughout, as an index of cognitive effort. We compared activity to RVIP task hits to hits on a control task (with similar visual parameters and response requirements but no working memory load): this contrast showed activity in medial frontal, inferior and superior parietal, temporal and visual cortices, consistent with previous work, demonstrating that meaningful neural data can be extracted from the RVIP task over an extended interval and using an event-related design. Behavioural performance was not affected by genotype; however, a genotype by condition (experimental task/control task) interaction on pupil diameter suggested that ε4 carriers deployed more effort to the experimental compared to the control task. fMRI results showed a condition by genotype interaction in the right hippocampal formation: only ε4 carriers showed downregulation of this region to experimental task hits versus control task hits. Experimental task beta values were correlated against hit rate: parietal correlations were seen in ε4 carriers only, frontal correlations in non-carriers only. The data indicate that, in the absence of behavioural differences, young adult ε4 carriers already show a different linkage between functional brain activity and behaviour, as well as aberrant hippocampal recruitment patterns. This may have relevance for genotype differences in cognitive ageing trajectories.

## Introduction

In this study we investigated cognitive performance and neural activation differences in young adult carriers of the Apolipoprotein E (*APOE*) ε4 allele using the rapid visual information processing (RVIP) task [1], a paradigm frequently used to measure sustained attention. The *APOE* ε4 allele is a well-established risk factor for Alzheimer’s disease (AD) [2] which also negatively impacts healthy cognitive aging [3]. In young adulthood, some studies have found task-specific cognitive *advantages* in ε4 carriers: previous work using the RVIP task found that ε4 carriers (henceforth referred to as e4+) outperform their non-ε4 peers (e4-) over a 6-minute version of the task [4, 5]. The RVIP task requires sustained attention to a digit stream(numerals 1–9) presented at a rate of 80/min. Participants respond when they detect a ‘target’ (a sequence of either 3 odd (e.g. 3, 7, 1), or 3 even (e.g. 4, 8, 2) digits) embedded within the digit stream. Typically, eight target sequences are presented per minute of the task. The task is well-established as a paradigm sensitive to fatigue, to fatigue-modifying drugs [6–8] and to cognitive ageing [9]. Only a small number of studies have examined its neural underpinnings using fMRI [10, 11]; others have combined fMRI with a nicotine manipulation [6, 12]: importantly, these studies have used block designs which constrains the epoch over which the task can be run. These studies contrasted RVIP task blocks against interleaved control task blocks (employing the same stimuli but with the last digit of each target sequence replaced by a ‘0’: participants are instructed to simply respond to the ‘0’, thus eliminating the working memory requirement). Block designs limit the task duration due to the requisite temporal filtering; Neale et al. [11], for example, used repeated interleaved blocks of only 1 minute each. In contrast, the standard behavioural paradigm presents the RVIP experimental task over a much longer, sustained, single interval (typically 5 to 10 minutes) and is thus far more attentionally demanding, with a key index being the performance decrement over time [1, 4, 5]. Importantly, the imaging studies report no decrement in performance as the session progresses, with control task performance at ceiling [6, 11]. Thus, blocked designs are unable to capture the deterioration in performance usually seen over several minutes of continuous RVIP performance, and are therefore unlikely to be able to capture the ε4 effects on sustained attention noted above. Indeed, Neale et al [11] note that “imaging paradigms of the RVIP task might be better characterised as tasks of ‘pure’ rather than ‘sustained’ attention”. These studies have shown that RVIP task blocks (contrasted against control blocks) activate middle frontal, inferior and superior parietal regions [10, 11]. Lawrence et al. [10] correlated neural activity against performance, showing that a better hit rate is associated with activation in right fronto-parietal networks, and with increased deactivation in left temporo-limbic areas. Neale et al.[11] additionally conducted an event-related analysis (where ‘hits’ (target detections) were modelled as events, and experimental hits contrasted against control hits as an index of vigilance-related activity). Encouragingly, there was a high degree of overlap between block- and event-related analyses. Commonalities (task>control) were observed in bilateral frontal, left parietal, thalamus and cerebellum; and (control>task) in occipital lobe. This was the first time an event-related approach had been tried: previously, authors had reservations about whether an event-related analysis could capture the neural activity reflecting the sustained nature of the vigilance requirements. However, Neale et al.’s findings suggest that vigilance-related activity can be extracted in an analysis that only models hits.

Since targets are randomly interspersed over the task, this opens the possibility of imaging the task over an extended interval, as intended in its original formulation as a test of sustained attention. For the purposes of this study, it was important that we examine sustained attention over an extended task interval for consistency with previous behavioural investigations showing that young adult e4+ sustain a higher level of performance (in terms of a higher hit rate) over a 6-minute task epoch [4, 5], with some evidence of a time by genotype interaction [4]. We therefore designed the session to comprise a 6 minute experimental task (as employed previously outside the scanner [4, 5]), plus a 6-minute control task (respond to ‘0’). As well as being the first fMRI investigation of *APOE* effects on sustained attention, to our knowledge this was the first time the RVIP task has been imaged over an extended interval. In order to validate this approach, imaging results were compared in detail against those reported previously from block designs (Neale et al. [11], Lawrence et al. [10]). Initial analyses contrasted hits against baseline for experimental and control tasks, separately (for comparison with Neale et al. [11]). Then, a contrast was employed to identify regions more active during experimental task hits than control task hits. Beta values were then extracted from key regions and correlated against performance (for comparison with Lawrence et al. [10]).

The cognitive and neural effects of the *APOE* gene have been subject to intense study in recent years, on the basis that carriers of the ε4 variant are at considerably higher risk of developing late onset Alzheimer’s disease (AD) [2] and also show poorer cognitive ageing in non-clinical populations [13]. Interestingly, ε4 effects on neural activity seem to be detectable relatively early in the lifespan. Studies have found evidence that e4+ show different patterns of neural activation relative to (e4-) in young adult populations. In a previous study using a covert attention paradigm, Rusted et al [5] reported that e4+ show greater activity in parietal regions relative to e4-; conversely, previous work with a prospective memory task found that e4+ show significantly less medial frontal activity during task trials [14]. We therefore predicted similar genotype effects in the present study.

Functional imaging studies investigating ε4 effects have often used memory tasks to probe activity within the medial temporal lobe (MTL), as this region is amongst the first to show evidence of AD pathology [15]. Evidence points to a pattern of hippocampal/parahippocampal overactivity in young adult e4+: we [16] and others [17] have shown this during the acquisition phase of subsequent memory paradigms. Notably, such overactivity in young adult e4+ has also been reported during tasks that would not be expected to recruit hippocampal regions, such as a Stroop task [18], and a covert attention task [5]. Young adult e4+ also show enhanced co-activation within hippocampal [19] and default mode [20, 21] resting state networks. These findings have prompted some authors to argue that this overrecruitment of hippocampal regions by e4+ could reflect compensatory activity in response to preclinical AD changes, and might also be the source of task performance differences [22]. Studies in healthy older e4+ [23–26] have also found MTL overactivity, often linked to task performance and therefore supporting a compensatory account. Reduced structural volumes within MTL has been reported in e4+, even in young adulthood [27, 28], supporting such an assertion. However, some conflicting results in young adults complicate this interpretation: Mondadori et al. [29] used an associative memory task and found that only e4+ showed performance-linked *decreases* in hippocampal recruitment as the task progressed, and Evans et al [16] reported that while e4+ overrecruit parahippocampal regions during subsequent memory acquisition, *under*-recruitment was observable during recall. Although a compensatory account might therefore be overly simplistic in this instance, hippocampal/parahippocampal activity in young adult e4+ certainly seems to be aberrant across a range of task conditions.

Work investigating effects of e4 in mid age populations also points to MTL as a crucial locus: Salvato et al. [30], using a memory-guided attention paradigm (which specifically engages the hippocampus [31, 32]), found that possession of the e4 allele undermines memory-based facilitation of attention in individuals with a mean age of 45. Since a similar effect was observed in healthy older individuals [33], this suggests that e4+ might evidence accelerated neural ageing. Our previous work in mid-age e4+ is also consistent with this interpretation [34]. On the basis of these published reports, we specifically predicted *APOE* functional activity differences in MTL. This study is the first to investigate *APOE* functional activity differences using a sustained attention paradigm. Results will be of interest with regard to the aberrant nature of MTL activity in young adult e4+. As such, a region of interest approach was taken, applying a small volume correction using an anatomical mask comprising hippocampal and parahippocampal regions.

We also used pupillometry to further investigate the notion of compensatory recruitment in e4+. Compensatory recruitment accounts suggest that e4+ devote greater resources to cognitive tasks in response to pathological changes [35]; this compensation could reflect the deployment of enhanced cognitive effort to maintain performance [23]. Thus, here we measured cognitive effort using pupillometry, to investigate whether differences in neural recruitment and performance might be attributable to increased cognitive effort in e4+. Pupil size has been linked to cognitive effort across various cognitive domains (subsequent memory [36], sentence comprehension [37], pitch discrimination [38]), and divided attention [39]. Studies also suggest that pupillometry measures could index AD risk: participants with mild cognitive impairment (MCI) show greater pupil diameter during digit recall, indicating compensatory effort to maintain performance compared to healthy controls [40]. We collected pupil size measurements during the task, to investigate whether enhanced behavioural performance in e4+ might be linked to greater pupil diameter, and thus greater deployment of cognitive effort.

In this study we set out to define activation patterns to task hits in the RVIP task, a test of sustained attention. Using event-related fMRI and a 6-minute task interval, this study contrasts with previous imaging investigations of the task that employed a block design and therefore a much more limited task interval. The extended task interval allowed us to investigate potential performance and neural recruitment differences associated with the *APOE* ε4 allele. Task-related activity was well matched with previous investigations, validating this approach. Interesting genotype effects emerged in parahippocampus, a region strongly implicated by previous neuroimaging investigations of *APOE* ε4 in young adults.

## Materials and methods

### Participants

We recruited a database of 328 healthy participants (aged 18–28 years) from the University of Sussex. Human Tissue Act protocols and informed consent procedures were followed throughout: participants consented to not being informed of their genotype. *APOE* genotype was determined by buccal swab. Genotype analyses were performed commercially (LGC Genomics, Hoddesdon, UK) using their system of fluorescence-based competitive allele-specific polymerase chain reaction (KASPar) targeting two *APOE* single-nucleotide polymorphisms (SNPs): rs429358 and rs7412. Participant call-back was performed blind, genotypes were added to datasheets by a 3^rd^ party after data was anonymised and preprocessed. Study invites were based on a random sampling so genotype status could not be inferred from an invitation to participate. Of the 328 participants who provided a buccal swab, 61 volunteers were found to carry at least one e2 allele and were excluded. 69 volunteers carried at least one ε4 allele: of these, 40 were invited to the study, of which 27 consented to take part. 197 volunteers were found to be homozygous e3 carriers, of these 50 were invited to the study, 26 of these consented to take part. The e4+ group contained 6 homozygous ε4 carriers. Inclusion criteria were: age 18–28, right handed, and fluent English speaker. Participants were excluded if they reported having high blood pressure, current treatment for a psychiatric condition, or failed the MRI safety screening. Volunteer characteristics are reported in Table 1. The two genotype groups were no different in age (1-way ANOVA, F = 0.003, p = 0.953). The total sample consisted of 21 males and 32 females, the proportion of females was not significantly greater than 50% (1 tailed proportion test, z = 1.385, p = 0.083). Gender balance was not significantly different between genotype groups (Chi-squared test, Chi-squared = 0.713, p = 0.399).

**Table 1:** Volunteer characteristics.

All participants		
Group	Age (years)	Gender
e4- (n = 26)	21.00 ± 2.00	14F/12M
e4+ (n = 27)	21.04 ± 2.64	18F/9M
F-statistic	0.003, ns	

### Experimental design

All participants volunteered under a written informed consent procedure approved by the Sussex University Schools of Psychology and Life Sciences Research Ethics Committee. Experimental procedures complied with the Code of Ethics of the World Medical Association (Declaration of Helsinki). The task was run as a component of a one hour scanner session.

**Fig 1:**
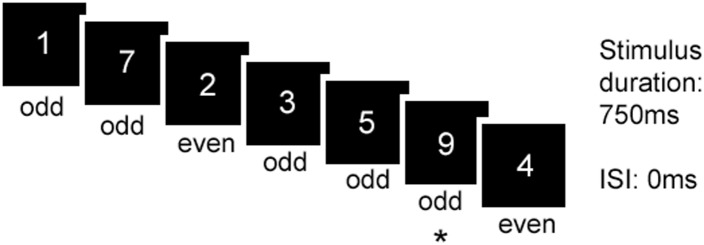
RVIP experimental task. Participants monitor a continuous stream of digits and respond when they have detected 3 odd or 3 even numbers in a row. In this example, a correct response is marked as (*).

The RVIP task was programmed in E-Prime v 2.0, and consisted of a control and experimental task, the order of which was counterbalanced. Structural imaging took place for around 20 minutes in between the two RVIP tasks. The RVIP experimental task lasted 6 minutes and consisted of monitoring a continuous stream of single digits (1–9) on a computer screen, which were presented at a rate of 80 digits per minute (each digit on-screen for 750ms, Fig 1). The digit stream was the same for all participants. Participants were instructed to press a button when they detected either three even or three odd numbers in a row. Eight of these target sequences occurred per minute, and correct detections were recorded within a 1500ms window following onset of the last digit in the target sequence. The control task consisted of an identical stream of digits, except that third digit in each target sequence was replaced with a zero, and participants were instructed to simply respond when they saw a zero. Thus, the control task was matched in terms of visual properties and response requirements, but without a working memory load. Participants practised the experimental task prior to scanning: a sequence containing two targets (one of odd numbers, one of even numbers) was presented; participants repeated the practise until they successfully identified both targets.

For experimental and control conditions, number of correct detections (hits), average latency to correct detections, and the number of false alarms (responses to nontargets) were recorded and averaged over each 1-minute segment of the task. Data analysis was conducted in SPSS. Data was checked to determine whether assumptions for use of parametric tests were satisfied; a repeated measures ANOVA was subsequently performed. The within-subject variable was time (6 levels, corresponding to each 1-minute segment), between subject variable was genotype (2 levels, e4+ and e4-). Task order (whether a participant completed the experimental or control task first), and gender, were entered as covariates. Analyses showed no main effects or interactions with task order in any of the data; similarly no main effects or interactions with gender were found: nevertheless these factors were included as covariates in all analyses.

### fMRI recording and analysis

fMRI datasets were acquired at 1.5 T (Siemens Avanto, 32-channel head coil, SQ-engine gradients) using a T2* -weighted 2D-EPI sequence To minimise signal artefacts originating from the sinuses, axial slices were tilted 30° from inter-commissural plane. Thirty-six 3 mm slices were acquired (0.75 mm interslice gap, interleaved (ascending) acquisition, phase encoding direction = A/P) with a matrix size of 64 and an in-plane resolution of 3 mm × 3 mm (TR = 3300 ms per volume, TE = 50 ms, flip angle = 90°, FoV = 192mm). A total of 118 volumes were acquired (including 5 dummy volumes) yielding a total acquisition time of ~ 6.5 minutes. Field maps (phase and magnitude images) were also acquired for use in the unwarping stage of data preprocessing. Participants were positioned comfortably within the head coil; foam padding around the head minimized head movements. The experimental protocol was viewed on a screen via a mirror attached to the head coil. An MR compatible button box was used to collect responses: participants indicated a target detection using the first finger of their right hand.

Images were pre-processed using SPM8 (RRID:SCR_007037) (http://www.fil.ion.ucl.ac.uk/spm/). Default settings were used throughout. After discarding the first 5 volumes, the remaining volumes were spatially realigned to correct for head motion, and unwarped. A mean image created from the realigned volumes was spatially normalized into standard stereotaxic space (at 3 × 3 × 3 mm^3^) using the Montreal Neurological Institute (MNI) template in SPM8. The derived spatial transformation was then applied to the realigned and unwarped volumes, which were finally spatially smoothed to facilitate group level statistics with a Gaussian kernel of 8-mm FWHM. fMRI data were analysed with the standard hierarchal model approach employed in SPM. Design matrices were constructed separately for each participant’s control and experimental phase. Events were modelled as events of zero duration (‘Hits’, ‘Misses’, and ‘False Alarms’): Thus each design matrix comprised 3 regressors of interest, plus subject-specific realignment-parameters and the session constant as effects of no interest. SPM8’s default anatomical hemodynamic response function was employed and effect sizes estimated for each regressor in the design matrix within the framework of the generalised linear model. So as to assess activity associated with successful target detections, 2 directional t-contrasts were estimated for each participant individually at the first level, corresponding to ‘Hits’ greater than/less than the implicit baseline (ie. all unmodelled events: task events not classified as a ‘Hit’, ‘Miss’ or ‘False Alarm’). This was conducted separately for each participant’s control and experimental phase. Resultant contrast images from each phase were taken to the second level. Full factorial ANOVA was used to assess ‘Hit’ related activity within each phase, and effects of condition (experimental/control) and genotype (e4-/e4+), gender and session order were included as covariates. For all comparisons, a FWE-corrected threshold of p < 0.05 was applied. For main task effects, correction was performed over the whole brain. For genotype effects, given our a priori hypothesis, a small volume correction was applied using a mask defined according to the automated anatomical labeling atlas (AAL) from the Wake Forest University PickAtlas toolbox (RRID:SCR_007378) [41]. This mask comprised the bilateral hippocampi and parahippocampi. To investigate correlations between activity and behaviour, relevant beta weights were extracted using the MarsBaR toolbox (RRID:SCR_009605).

### Pupillometry recording and analysis

Pupil diameter was recorded throughout the fMRI acquisition using an ASL Eyetrac 6 system with a 120Hz sampling rate. Data was converted using ASL’s EyeNal software package (RRID:SCR_005997). Data was quality checked and deemed usable for 43 participants (20 e4+ and 23 e4-). The criteria for including a participant was that >75% of data samples had to be available. Intermittent tracking of the pupil, resulting in insufficient data samples, was due to use of the MRI-safe goggles, light-coloured irises, or head position in the coil. For each participant, pupil diameter was averaged across each minute of the task. The rapid presentation of stimuli compared to the relatively slow dynamic response of the pupil precluded a more fine-grained analysis (e.g. calculating pupil diameter to successful target detections).

## Results

### Task performance—Rapid Visual Information Processing (RVIP)

One volunteer was excluded from the e4+ group due to poor performance on the control task (accuracy for correct detections was 52%). For the remaining volunteers, correct detection rate, latencies and false alarms for the experimental and control tasks are presented in Table 2.

**Table 2:** RVIP task performance.

	Experimental Task			Control Task		
Group	Mean detections per min (max 8)	Mean false alarms per min	RT (ms) to correct detections	Mean detections per min (max 8)	Mean false alarms per min	RT (ms) to correct detections
e4- (n = 26)	5.61 (1.28)	0.57 (0.45)	535.36 (41.39)	7.78(.63)	0.032(.067)	471.40 (55.79)
e4+ (n = 26)	5.33 (1.04)	0.77 (0.68)	551.54 (57.92)	7.92(.14)	0.051(.11)	462.93 (34.34)
F stat. (p value)	0.760 (0.388)	1.554 (0.218)	1.344 (0.252)	1.353 (0.250)	0.556 (0.460)	0.435 (0.513)

#### Experimental task

**Fig 2:**
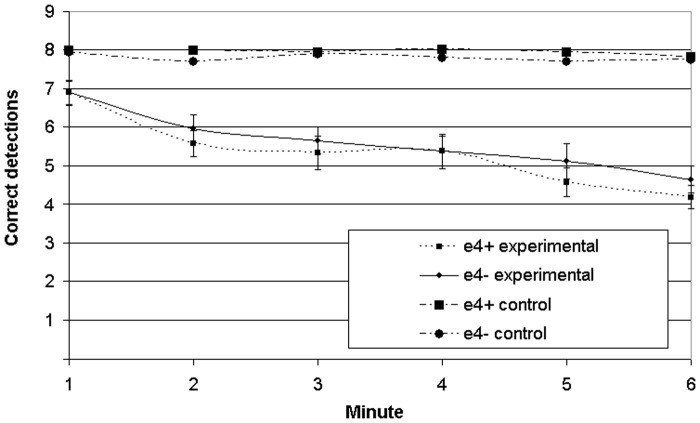
Number of hits, by minute, for experimental and control tasks. F and p values for comparison between groups.

**Correct detections of target sets**. There was a main effect of time on correct detections (F(5, 240) = 23.230, p<0.001). There was no main effect of genotype (F(1, 48) = 0.760, p = 0.388) and no time by genotype interaction (F(5, 240) = 0.435, p = 0.824). These data are presented in Fig 2.

**RTs to correct detections**. There was a main effect of time on latency (F(5, 240) = 3.736, p = 0.003), with RTs increasing as the task progressed. A paired-samples t-test compared RTs over minute 1 (mean = 500ms, s.d. = 64.4ms) to RTs over minute 6 (mean = 551ms, s.d. = 93.8ms): a significant difference was found (t(51) = -3.45, p = 0.001) indicating a slowing of RTs from the first to last minutes of the task. There was no main effect of genotype (F(1, 48) = 1.344, p = 0.252) and no time by genotype interaction (F(5, 240) = 0.464, p = 0.823).

**False alarm rate**. There was a main effect of time (F(5, 240) = 5.543, p<0.001), with mean false alarm rate increasing between minute 1 (mean = 0.615, sd = 0.911) and minute 6 (mean = 1.153, sd = 0.894); tested using a paired-samples t-test (t(51) = -3.34, p = 0.002). There was no main effect of genotype (F(1, 48) = 1.554, p = 0.218) and no time by genotype interaction (F(5, 240) = 1.217, p = 0.301).

#### Control task

**Correct detections of target sets**. There was no main effect of time on correct detections (F(5, 240) = 1.523, p = 0.183), and no main effect of genotype (F(1, 48) = 1.353, p = 0.250). There was no time by genotype interaction (F(5, 240) = 1.028, p = 0.401).

**RTs to correct detections**. There was a main effect of time on latency (F(5, 240) = 7.536, p<0.001). RTs over minute 1 (mean = 460ms, s.d. = 48.2ms) were significantly faster than over minute 6 (mean = 485ms, s.d. = 60.1ms), tested by paired-samples t-test (t(51) = -4.76, p < 0.001). There was no main effect of genotype (F(1, 48) = 0.435, p = 0.513) and no time by genotype interaction (F(5, 240) = 1.314, p = 0.259).

**False alarm rate**. There was no main effect of time (F(5, 240) = 1.945, p = 0.087), no main effect of genotype (F(1, 48) = 0.556, p = 0.460), and no time by genotype interaction (F(5, 240) = 0.468, p = 0.800).

### Pupillometry data

Linear mixed modelling was used to investigate the effects of genotype and RVIP condition on pupil diameter. Repeated measures were specified as minute and task condition, and compound symmetry was utilised as the repeated covariance type. The model included main effects of time, genotype and RVIP condition, and interaction effects. When a random effect of participant was included in the model the covariance parameter was reported as redundant and so was excluded from further analyses. Results were corrected for multiple comparisons (Bonferroni). Means and standard deviations are reported in Table 3. There was a significant main effect of condition (F(1, 451) = 34.977, *p* < 0.001), with pupil diameter greater in the experimental task than in the control task. There was no significant main effect of genotype in either the experimental task (F(1,41) = 0.402, p = 0.530) or the control task (F(1,41) = 0.348, p = 0.558). There was no main effect of time.

**Table 3:** Pupillometry data across different RVIP conditions. Values represent the mean with standard errors in brackets; units are arbitrary.

	Mean pupil diameter (standard error)	
Group	Experimental Task	Control Task
e4- (n = 23)	37.57 (1.41)	36.64 (1.33)
e4+ (n = 20)	38.72 (1.38)	35.72 (1.04)

**Fig 3:**
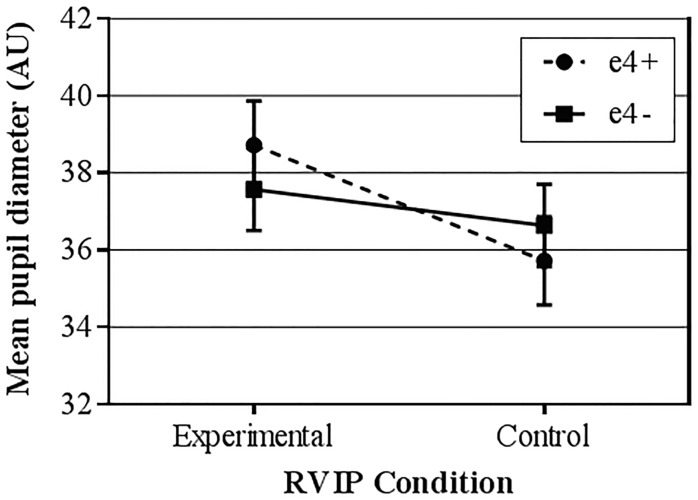
Mean pupil diameter by RVIP condition separated by genotype. Error bars represent the standard error; units are arbitrary.

There was a significant interaction between genotype and RVIP condition (F(1, 451) = 9.636, *p* = .002; Fig 3). RVIP condition strongly affected e4+ (F(1, 209) = 43.715, *p* < .001) while in e4- it was close to significant (F(1, 242) = 3.815, *p* = .052). There were no interactions with time.

### fMRI data

#### Experimental task

**T-contrast: Hits > baseline**. Activations to successful detections during the experimental task were investigated relative to the implicit baseline (ie. all unmodelled events: hits, misses and false alarms were included in the design matrix). Significantly greater activity was observed in regions including frontal, middle cingulate, inferior parietal, visual cortex and cerebellum (Table 4).

**Table 4:** Regions where activity to experimental task hits was greater than the implicit baseline.

Region	Vox	X, y, z	Sig
**Left insula**	**1976**	**-24, 2, 0**	**P<0.001 FWE-corrected (cluster—level)**
**Medial Frontal**	**2434**	**-2, 46, 12**	**P<0.001 FWE-corrected (cluster—level)**
**Right middle temporal gyrus**	**339**	**66, -20, 18**	**P = 0.002 FWE-corrected (cluster—level)**
**Cerebellum**	**299**	**26, -56, -22**	**P = 0.003 FWE-corrected (cluster—level)**
**Left inferior parietal cortex**	**184**	**-52, -32, 54**	**P = 0.008 FWE-corrected (cluster—level)**
**Left BA17/BA18**	**232**	**-22, -92, 0**	**P = 0.002 FWE-corrected (cluster—level)**
**Cerebellum**	**336**	**-4, -82, -22**	**P<0.001 FWE-corrected (cluster—level)**
**Middle cingulate**	**589**	**2, -8, 30**	**P<0.001 FWE-corrected (cluster—level)**
**Right inferior parietal cortex**	**272**	**48, -56, 40**	**P = 0.001 FWE-corrected (cluster—level)**
**Right inferior frontal**	**443**	**44, 48, -10**	**P<0.001 FWE-corrected (cluster—level)**
**Right insula**	**235**	**24, 12, -12**	**P = 0.002 FWE-corrected (cluster—level)**
**Cerebellum**	**196**	**-36, -82, -20**	**P = 0.005 FWE-corrected (cluster—level)**

**T-contrast: Baseline > hits**. No significant effects were found.

#### Control task

**T-contrast: Hits > baseline**. Significantly greater activity to successful detections during the control task relative to baseline was observed in cerebellum (p<0.001 FWE—corrected, 2756 vox, 4, -80, 20).

**T-contrast: Baseline > hits**. No significant effects were found.

#### Experimental/control task comparisons

**Fig 4:**
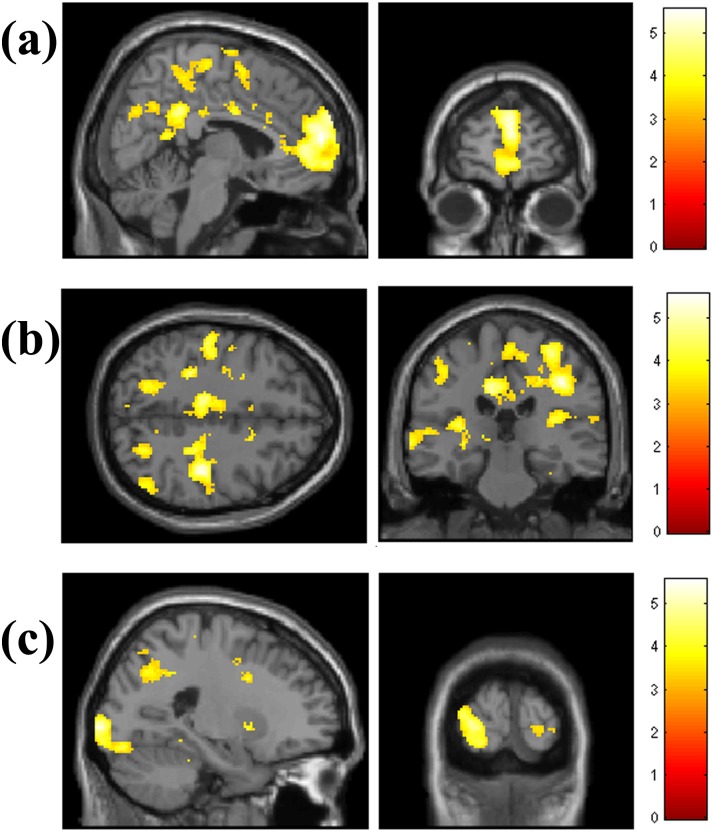
Experimental task hits > control task hits in (a) medial frontal (X = 6.24mm, Y = 61.5mm) and (b) bilateral IPC/middle cingulate (Z = 36.2mm, Y = -28.7mm) and (c) visual cortex and SPL (X = -21.4mm, Y = -92.1mm).

**T-contrast: Hits (experimental) > hits (control)**. Activity in medial frontal (Fig 4a), inferior and superior parietal, temporal and visual cortices was seen to be greater during experimental task hits compared to control task hits (Table 5). The bilateral IPC cluster spanned both hemispheres and incorporated middle cingulate (Fig 4b).

**Table 5:** Regions where activity to experimental task hits was greater than control task hits.

Region	Vox	X, y, z	Sig
**Medial Frontal**	**2833**	**4, 58, 12**	**P<0.001 FWE-corrected (cluster—level)**
**Bilateral IPC/middle cingulate**	**5809**	**40, -26, 40**	**P<0.001 FWE-corrected (cluster—level)**
**Left visual cortex (BA18)**	**848**	**-22, -100, 0**	**P<0.001 FWE-corrected (cluster—level)**
**Right middle temporal**	**385**	**64, -4, 20**	**P = 0.001 FWE-corrected (cluster—level)**
**Left Insula**	**1162**	**-34, -24, 12**	**P<0.001 FWE-corrected (cluster—level)**
**Left visual cortex**	**269**	**-36, -62, -14**	**P = 0.005 FWE-corrected (cluster—level)**
**Right visual cortex**	**242**	**28, -88, 26**	**P = 0.009 FWE-corrected (cluster—level)**
**Left SPL**	**202**	**-18, -60, 38**	**P = 0.020 FWE-corrected (cluster—level)**
**Right SPL**	**282**	**26, -64, 34**	**P = 0.004 FWE-corrected (cluster—level)**
**Right inferior parietal cortex**	**200**	**52, -64, 40**	**P = 0.021 FWE-corrected (cluster—level)**

**Hits (control) > hits (experimental)**. No significant effects were found.

### Correlations with behaviour

To investigate the behavioural significance of activity in key regions where activations differentiated task hits from control hits, an exploratory analysis was conducted. Beta values were extracted for each participant (10mm sphere centred on peak voxel) and correlated against behavioural measures (hit rate, RT) for the experimental task. Correlations were assessed across all volunteers, separately for each genotype (Table 6). Across all participants, activity in medial frontal correlated positively with hit rate, which was extremely close to significance (p = 0.050). Neither of the other regions showed any correlation. In e4-, the correlation in medial frontal was significant (p = 0.01), no relationship was seen in e4+ (p = 0.997). Tested using a Fisher r-to-z transformation, the difference between these correlations was significant: Z = 1.85, p = 0.03 (1-tailed). Previous findings suggested a diminished medial frontal response to task trials in e4+ [14], justifying use of a 1-tailed test. Conversely, in bilateral IPC, a strong trend towards a positive correlation between activity and hit rate was evident in e4+ (p = 0.0559); this was not present in e4- (-ve correlation, p = 0.563). However, it should be noted that these results are not corrected for multiple comparisons: as such, findings should be interpreted with caution.

**Table 6:** Correlations between beta values and behavioural measures in medial frontal, bilateral IPC and left Insula; r values (p values in brackets).

Region	All subjects		E4-		E4+	
	Hits	RT	Hits	RT	Hits	RT
**Medial Frontal**	**.273(.050)**	**-.042(.767)**	**.493(.010)**	**-.162(.430)**	**.001(.997)**	**.018(.929)**
**Bilateral IPC/precuneus/middle cingulate**	**.101(.475)**	**-.243(.083)**	**-.119(.563)**	**-.265(.191)**	**.379 (.0559)**	**-.195(.340)**
**Left Insula**	**.022(.877)**	**-.092(.518)**	**.157(.444)**	**-.244(.229)**	**-.090(.662)**	**-.029(.888)**

### Effects of *APOE* genotype

#### Main effect of genotype

Main effects of genotype were assessed for all the contrasts described above. No significant effects were found.

#### Genotype by condition interaction

**Fig 5:**
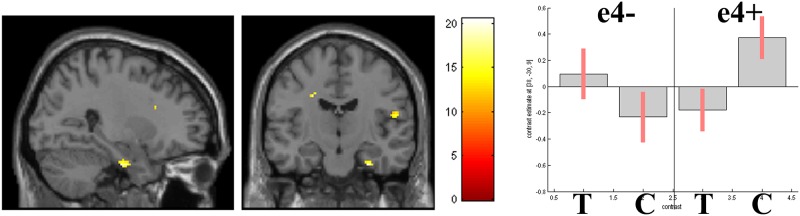
Genotype by condition interaction in the right hippocampal formation. Render (X = 28mm, Y = -14mm, at p<0.001 unc.) and parameter estimates (and 95% CI) for each genotype group during Experimental Task hits (T) and Control Task hits (C).

In the model comparing task and control hits, a significant genotype by condition interaction was observed, after SVC for hippocampus/parahippocampus (based on a priori hypotheses). A significant cluster was observed in the right hippocampal formation, localised to the subiculum (30 vox, p = 0.024 FWE-corrected, 28, -14, 30). Parameter estimates suggested that the interaction was driven by greater activity to control hits over task hits in e4+ (Fig 5). To assess this, the interaction was interrogated using a specific T-contrast: Control hits>Experimental hits (e4+ only). After SVC, this t-contrast identified a cluster which approached significance (22 vox, p = 0.067 FWE-corrected, 30, -14, 30).

In an exploratory analysis to assess the behavioural significance of this interaction, beta weights during task hits and control hits were extracted using the Marsbar toolbox, for each participant, using a 5mm sphere around the peak voxel (28, -14, 30). Beta weights were then correlated against the behavioural measures. Betas from the experimental task hits were correlated against Hit rate, RT and number of false alarms during the experimental task. No significant correlations were found. Similarly, betas from the control task hits were correlated against number of hits, RT and number of false alarms during the control task. Significant correlations were observed between beta values and false alarms (r = -0.277, p = 0.047) and RT (r = -0.367, p = 0.007). Separated by genotype, the correlation with false alarms was significant only in e4+ (e4+, r = -0.492, p = 0.011; e4-, r = -0.094, p = 0.649), while the correlation with RT was significant only in e4- (e4-, r = -0.541, p = 0.004; e4+, r = 0.036, p = 0.860).

## Discussion

In this study we used fMRI to investigate activation patterns associated with a 6-minute sustained attention task. In contrast with previous imaging studies of the RVIP task, we did not employ an interleaved block design that limits the duration of the sustained attention interval. The 6-minute task has the advantage of being a ‘true’ test of sustained attention, being consistent in design with the original formulation of the task (Wesnes & Warburton, 1983 [1]). Previous studies have avoided event-related approaches over concerns that an event-related model would not be sensitive to sustained attention processes. Studies examining task>control effects in a block design have reported activity bilaterally in inferior and superior parietal, inferior and middle frontal, pre-SMA, thalamus, caudate, anterior insula and cerebellum (Lawrence et al. [10]; young adult sample, mean age 24), and bilateral middle frontal, right superior/inferior parietal, and right thalamus (Neale et al. [11]; mid age sample, mean age 54). Critically, Neale et al. [11] compared block and event-related analyses within an interleaved block design and found a high degree of overlap in results. This validation supported our event-related approach and we saw good correspondence between our results and previous work employing block designs. Experimental and control hits compared to their respective baselines showed similar activation patterns to those reported by Neale et al. [11]. Experimental hits over baseline (implicit baseline incorporating all unmodelled task events) showed activity in various frontal regions as well as middle cingulate, inferior parietal, visual cortex and cerebellum; Neale et al. [11] report activity in a similar network when task was contrasted against an explicit baseline (although medial frontal activity was more prominent in the current study). Most importantly, activity to experimental task hits contrasted against control task hits overlapped substantially with previous work. Neale et al. report that contrast as activating medial frontal, precuneus, posterior lobe and inferior parietal regions. In this study, we also found the most substantial clusters of activation to be in medial frontal, inferior parietal and visual cortex on this contrast. We also found activity in superior parietal, left insula, and right middle temporal regions. Lawrence et al, [10] also reported greater activity in left insula (alongside inferior/superior parietal, inferior/middle frontal) during experimental task blocks compared to control blocks. These high levels of correspondence between regions activated in previous designs and the event–related approach employed here suggests that an event-related model can adequately capture the attentional processes subserving the task. The task can therefore be imaged as originally formulated, taxing sustained attention over an extended interval. As noted by Lawrence et al. [10], working memory processes also likely contribute to the activity observed (particularly in the fronto-parietal network, and insula). Block designs are unable to distinguish sustained attention from working memory processes; although beyond the scope of the current paper, the findings presented here point to the possibility of employing more complex analyses to model working memory load (e.g. contrasting isolated even/odd events against 2-odd/even in a row, and 3 odd/even in a row) so as to distinguish activity that is modulated by working memory demands from that attributable to sustained attentional processing.

Having first ascertained that task effects were consistent with previous studies employing block designs, this provided justification for use of this event-based design to explore previously reported differences in sustained attention performance in young adult e4+ [4, 5]. Lawrence et al. assessed the behavioural significance of activation in each cluster by examining correlations between beta values and RVIP experimental task performance (Hits and RT). We adopted the same approach, extracting beta values from key regions showing greater recruitment to experimental task versus control, and assessing correlations across all participants, and by genotype group. In left insula, no relationship was observed. Lawrence et al. likewise observed no correlations in a similarly located cluster (although that study also reported activity in a more anterior region, which showed a positive relationship with number of hits). In medial frontal, we found that activity correlated with task hits. Likewise, Lawrence et al. reported positive correlations with hits in medial frontal regions. In inferior parietal/precuneus/middle cingulate, we found a trend (p = 0.083) towards a negative relationship with RT; Lawrence et al. also reported a negative relationship with RT (and a positive relationship with hits) in a cluster that incorporated portions of inferior and superior parietal lobe.

Some interesting effects emerged when we explored correlations in each genotype group separately. A strong positive correlation between medial frontal activity and experimental task hits was seen in e4-, while e4+ showed no correlation at all. This difference was statistically significant, suggesting possible genotype-specific differences in how BOLD signal relates to performance, with frontal activation levels indexing task hits in e4- but not e4+. Conversely, e4+ showed a strong trend towards a positive correlation between activity in the parietal cluster and hits, whereas e4- showed no effects, although a comparison of r values between groups was not significant. These analyses should be regarded as exploratory and results interpreted with caution pending replication in larger samples (as the effects reported here would not survive correction for multiple comparisons), but there is consistency with previous findings. Although most previous *APOE* studies have focussed on effects in temporal structures, work investigating whole-brain volumetric differences has revealed lower gray matter density in bilateral frontal regions in e4+, across the lifespan [28]. Thinner frontal cortex has also been observed in mid age e4+, interpreted as reflecting susceptibility for poorer cognitive ageing [42]. We have previously reported that young adult e4+ show significantly less medial frontal activity to prospective memory task events, relative to e4- [14]. The present results, suggesting disrupted linkage between frontal activity and task performance, can be interpreted in this context. Also, greater activity in precuneus and posterior cingulate regions has been shown previously demonstrated in young adult e4+, during a scene perception and working memory task [43], and during a covert attention task [5]. Since AD-related metabolic changes manifest in posterior cingulate and precuneus early in the disease process [44], overactivation of these structures in early adult life could underlie subsequent neural changes linked to AD risk in e4+ [45]; in late mid-age, healthy e4+ evidence hypometabolism in these regions [46]. Thus, the current results, which potentially indicate greater task-related engagement of precuneus and neighbouring structures in young adult e4+, deserves further study.

Behaviourally, the two genotypes sustained equivalent hit rates. Previous studies found higher hit rates in e4+ [4, 5], further studies using larger samples of participants are required to clarify this finding. However, we identified genotype by condition (experimental/control task) interactions, in pupil diameter and hippocampal activity. Both of these interactions indexed greater difference between task and control conditions in e4+, suggesting that e4+ are more responsive to the cognitive demands of each task condition (here, working memory load differentiated experimental from control tasks). In pupil diameter, only e4+ showed significantly differences in pupil size between the task conditions, although e4- showed a strong trend in the same direction. This is interesting in the context of recent work showing greater pupil diameter (implying greater cognitive effort) in mild amnestic MCI patients during a memory task [40], compared to healthy controls. Performance differences were absent, and the results were interpreted as pupillometry indexing compensatory activity that allowed performance maintenance in the earliest stages of cognitive decline. More severe MCI patients did not show pupillometry differences, implying that ability to compensate had been exhausted in these individuals. Our pupillometry results, in a young adult sample at genetic risk of cognitive decline later in life, are analogous in that these individuals appeared to require greater cognitive effort to achieve equal levels of performance. This supports the notion that pupil diameter measurements could be sensitive to future risk of cognitive decline. Further work is needed to assess this possibility. This interpretation requires further exploration across other cognitive tasks and larger sample sizes.

The event-related fMRI analysis revealed a genotype by condition interaction in the right hippocampal formation (localised to the subiculum). This region is of interest since previous studies [5, 17, 18] have identified genotype differences in activation, although these have tended to be in the direction of e4+ overactivity. Here, we found that e4+ deactivated right hippocampus in response to the experimental versus the control task, although results were at trend level so should be interpreted with caution pending replication. Lawrence et al. [10] found that left parahippocampal regions were deactivated during task relative to control blocks, and better experimental task performance was associated with decreased activation. The results presented here suggest that, through enhanced hippocampal deactivation, and differential deployment of cognitive effort, e4+ might be using a different cognitive strategy. Indeed, hippocampal activity showed a different pattern of correlation with control task performance in e4+, supporting such an interpretation. Strategy differences, which might also be compensatory in nature, might provide the potential for performance advantages if appropriately deployed, but it is still not clear why performance differences in young ε4 carriers are inconsistently reported within as well as across cognitive domains.

### Conclusions

Results demonstrated the viability of imaging the RVIP task over an extended interval, using an event-related design. Activation patterns in response to task and control hits corresponded well with previous studies that employed block designs that likely compromise the sustained attention required by the task. Thus, future imaging investigations will benefit from using the RVIP task in its original (extended) form. On this basis, we investigated genotype effects on sustained attention performance and neural activity. Although task performance was comparable between e4+ and e4-, correlating beta values with performance suggested different approaches to the task demands: we observed a correlation between frontal activity and task performance only in e4-, consistent with previous findings of diminished frontal responses in e4+ [14]. The correlation findings (and it is important to note that these were not corrected for multiple comparisons) warrant further study in an expanded sample. Further, a genotype by condition interaction was evident in MTL, a structure where genotype effects on activation have previously been observed across a variety of paradigms [5, 17, 20, 29]. Again, replication in a larger sample would be useful, but e4+ appeared to deactivate MTL during the experimental task, suggesting that while MTL activity is likely aberrant in e4+, it is not necessarily compensatory in nature.

## Supporting information

S1 SPSS Summary Datasets (ZIP)
