# Supplementary material for: Using event-related fMRI to examine sustained attention processes and effects of APOE ε4 in young adults
Source: PLoS One. 2018 Jun 1;13(6):e0198312. doi: 10.1371/journal.pone.0198312 (PMC5983530; doi:10.1371/journal.pone.0198312)
Supplement: S1 SPSS Summary Datasets — (ZIP) [file pone.0198312.s001.zip › Datasets readme.doc]

**3 files of summary data**

**“Full behaviour by minute”** contains behavioural performance data.

Experimental task: ‘min#.hits’ is number of hits over minute # (where # = 1 through 6), ‘min#.falsa’ is number of false alarms (button press when no target was present) over minute #, and ‘min#.hitlat’ is reaction time (in msec) to hits over minute #.

Control task: ‘Cmin#.hits’ is number of hits over minute # (where # = 1 through 6), ‘Cmin#.falsa’ is number of false alarms over minute #, and ‘Cmin#.hitlat’ is reaction time (in msec) to hits over minute #.

**“Participant characteristics, summary pupil data, betas”** contains age, gender; mean pupil diameter for experimental task over minutes 1 to 6, and beta values extracted from the experimental task fMRI data for Medial Frontal (4, 58, 12), Bilateral IPC (40, -26, 40), Left Insula (-34, -24, 12), and Right Hippocampal formation (28, -14, 30). Beta weights during task hits were extracted using the Marsbar toolbox, for each participant, using a 5mm sphere around the peak voxel.

**“Full pupillometry data by minute”** contains mean pupil diameter, per minute, for both experimental task (“task”) and control task (“control”), by participant.

Participants are identified by “participant number” in all spreadsheets.

Genotype_group: denotes APOE group assignment where 3= e33, 4= e34 or e44

Gender: 0=male, 1=female

Session order: 1= Experimental task followed by control task, 0 = control task followed by experimental task
